# Prehospital identification of Covid-19: an observational study

**DOI:** 10.1186/s13049-020-00826-6

**Published:** 2021-01-06

**Authors:** Douglas Spangler, Hans Blomberg, David Smekal

**Affiliations:** Uppsala Center for Prehospital Research, Department of Surgical Sciences – Anesthesiology and Intensive care, Uppsala University, 751 85 Uppsala, Sweden

## Abstract

**Background:**

The novel coronavirus disease 2019 (Covid-19) pandemic has affected prehospital care systems across the world, but the prehospital presentation of affected patients and the extent to which prehospital care providers are able to identify them is not well characterized. In this study, we describe the presentation of Covid-19 patients in a Swedish prehospital care system, and asses the predictive value of Covid-19 suspicion as documented by dispatch and ambulance nurses.

**Methods:**

Data for all patients with dispatch, ambulance, and hospital records between January 1–August 31, 2020 were extracted. A descriptive statistical analysis of patients with and without hospital-confirmed Covid-19 was performed. In a subset of records beginning from April 14, we assessed the sensitivity and specificity of documented Covid-19 suspicion in dispatch and ambulance patient care records.

**Results:**

A total of 11,894 prehospital records were included, of which 481 had a primary hospital diagnosis code related to-, or positive test results for Covid-19. Covid-19-positive patients had considerably worse outcomes than patients with negative test results, with 30-day mortality rates of 24% vs 11%, but lower levels of prehospital acuity (e.g. emergent transport rates of 14% vs 22%). About half (46%) of Covid-19-positive patients presented to dispatchers with primary complaints typically associated with Covid-19. Six thousand seven hundred seventy-six records were included in the assessment of predictive value. Sensitivity was 76% (95% CI 71–80) and 82% (78–86) for dispatch and ambulance suspicion respectively, while specificities were 86% (85–87) and 78% (77–79).

**Conclusions:**

While prehospital suspicion was strongly indicative of hospital-confirmed Covid-19, based on the sensitivity identified in this study, prehospital suspicion should not be relied upon as a single factor to rule out the need for isolation precautions. The data provided may be used to develop improved guidelines for identifying Covid-19 patients in the prehospital setting.

## Introduction

### Background

The pandemic of novel coronavirus disease 2019 (Covid-19) caused by Severe Acute Respiratory Syndrome Coronavirus 2 (SARS-CoV-2) has impacted healthcare systems across the world, and much research has been done regarding the underlying mechanisms of the disease [1], the clinical presentation of affected patients [2, 3], prevention and treatment methodologies [4, 5], and the impact on population-level health [6]. Prehospital care providers play an important role in initiating disease isolation precautions and are instrumental in preventing contagion at ambulance receiving facilities. If prehospital care providers identify Covid-19 patients and document their suspicion with a high degree of precision, this documentation could be used as the basis for determining the need for isolation precautions on the ambulance and at receiving facilities.

Despite the significant Covid-19 related scientific output [7], only a few studies describing the effect of the Covid-19 pandemic on prehospital care systems are available [8–10], and these are largely limited to reports on the impact of the pandemic at the system-level. Only a single study has been published which quantitatively investigates the characteristics of Covid-19 patients and/or the ability of prehospital care providers to identify SARS-CoV-2-positive patients by linking prehospital data with subsequent hospital diagnoses [11]. In this study, Fernandez et al. found that Covid-19 patients more often presented to prehospital care providers with tachycardia, tachypnea, hypoxia, and fever, and found a sensitivity of 78% for ambulance crews in identifying confirmed Covid-19 patients based on an analysis of structured documentation and free text notes.

### Objective

In this study, we sought to describe the clinical presentation of Covid-19 patients in relation to appropriate comparison cohorts, and to assess the ability of prehospital care providers to identify Covid-19 patients the dispatch center and on the ambulance.

### Setting

The region of Uppsala, Sweden has an area of 8209 km2, and a population of 385,000. The region is served by one major university hospital and a smaller regional hospital, an ambulance service with 13/18 (night-time/daytime) ambulances, and a single regional Emergency Medical Dispatch (EMD) center. Ambulances are staffed by either two nurses, or one nurse and an emergency medical technician. Call-takers at the dispatch center are exclusively nurses, with 3–4 years of university education and a minimum of 3 years of clinical experience.

At the peak of the pandemic, the Emergency Medical Dispatch (EMD) center experienced a ca. 10–20% increase in call volume. The guidelines employed by the ambulance service and dispatch center to identify Covid-19 patients evolved over time as knowledge about the disease increased. The first guidelines regarding Covid-19 suspicion were implemented on February 28, 2020, setting the criteria for Covid-19 suspicion as the presence of a fever and respiratory symptoms (e.g., coughing or difficulty breathing), and having within the previous 14 days visited China, Italy, Iran, or South Korea, or been in contact with a person being evaluated for Covid-19. By March 13, community transmission of Covid-19 had been detected in Sweden, and the criterion based on previous travel was dropped. On April 1, guidelines were updated such that any one of four symptoms (Fever, cough, difficulty breathing, upper airway symptoms) was sufficient to trigger Covid-19 precautions. On May 7, diarrhea was added to the list of symptoms which would trigger Covid-19 suspicion.

The ability to document suspicion of Covid-19 in a structured format was added first to the dispatch clinical decision support system on March 19, and then to the ambulance’s electronic patient care reporting system on April 14. Prior to these dates, suspicion of Covid-19 was generally documented in free-text notes and/or communicated between care providers verbally via radio, telephone, or at patient handover.

## Methods

### Data sources

Data were extracted from dispatch, ambulance, and hospital records in the region of Uppsala, Sweden, and deterministically linked based on patient personal identification number and case identifier for the period of January 1–August 31, 2020. All single-patient EMD center contacts which could be linked to both an ambulance record and a hospital record were included in the study. For calls where the patient was evaluated but not transported by the ambulance, hospital records were searched, and patients visiting a healthcare facility within 72 h of contact with the prehospital care system were included in the analysis.

### Variables

Covid-19 patients were identified based on documentation of either a real-time reverse transcription positive polymerase chain reaction (PCR)-based test for an ongoing SARS-CoV-2 infection at any point during the first hospital care episode following contact with the ambulance, or a primary diagnosis code relating to Covid-19 (ICD-10-SE codes U07.1, U07.2). Samples for PCR testing were obtained using the combined oro-nasopharyngeal swabbing technique.

For the purposes of the descriptive analysis, patient cohorts were generated consisting of patients cared for prior to the beginning of the Covid-19 pandemic in Uppsala (January 1 – February 29), patients cared for at the hospital but not tested for SARS-CoV-2 (“Assumed negative”), patients tested and found negative for SARS-CoV-2 (“Confirmed negative”), and patients who tested positive or were otherwise diagnosed with Covid-19 (“Positive”).

Hospital outcome measures included rates of hospital admission, intensive care utilization, and 30-day mortality. Covariates extracted from dispatch and ambulance records for analysis included patient demographics (age and gender), response and transport priority (Lights and sirens vs. no lights and sirens), and initial ambulance findings (Airway/Breathing/Circulation assessments), vital signs collected by the ambulance (pulse, systolic blood pressure, breathing rate, Spo2, and temperature), and interventions performed by ambulance crews (Oxygen/medication administration). We included a variable indicating the primary complaint of the patient (as categorized by the dispatch nurse), and based on local guidelines, considered the complaints of infection, fever, difficulty breathing, and upper airway distress to be “typical” of Covid-19.

### Statistical methods

A time-series analysis was performed to investigate the impact of Covid-19 on the prehospital care system in the region over time, using local polynomial regression fitting (loess) on a back-transformed log(x + 1) scale to enable estimating rolling mean values at- and/or close to zero [12]. Statistics regarding means, medians, and percentages were generated as appropriate, using ordinary non-parametric bootstrapping to generate 95% confidence intervals [13]. Continuous variables with non-monotonic associations with Covid-19 status (e.g. vital signs) were described using kernel density plots. Missing vital sign data, and values above and below the 99.9th percentile were excluded from each respective plot in order to eliminate values stemming from documentation errors.

The accuracy of documented Covid-19 suspicion was assessed using standard epidemiological test statistics including sensitivity, specificity, and negative/positive predictive value. Confidence intervals for test statistics were calculated using exact binomial tests as implemented in the epiR package [14, 15]. Longitudinal shifts in predictive value were assessed by plotting each measure grouped by month. Given the potential for bias due to missed cases of Covid-19 among assumed negative patients, we analyzed statistics based both on the assumption that untested patients were negative per the study by Fernandez et al. [11], and a sensitivity analysis investigating only PCR-test results. All analyses were performed using R version 4.0.2 [16]. Results were reported based on the STROBE guidelines [17].

## Results

### Descriptive data

**Fig. 1:**
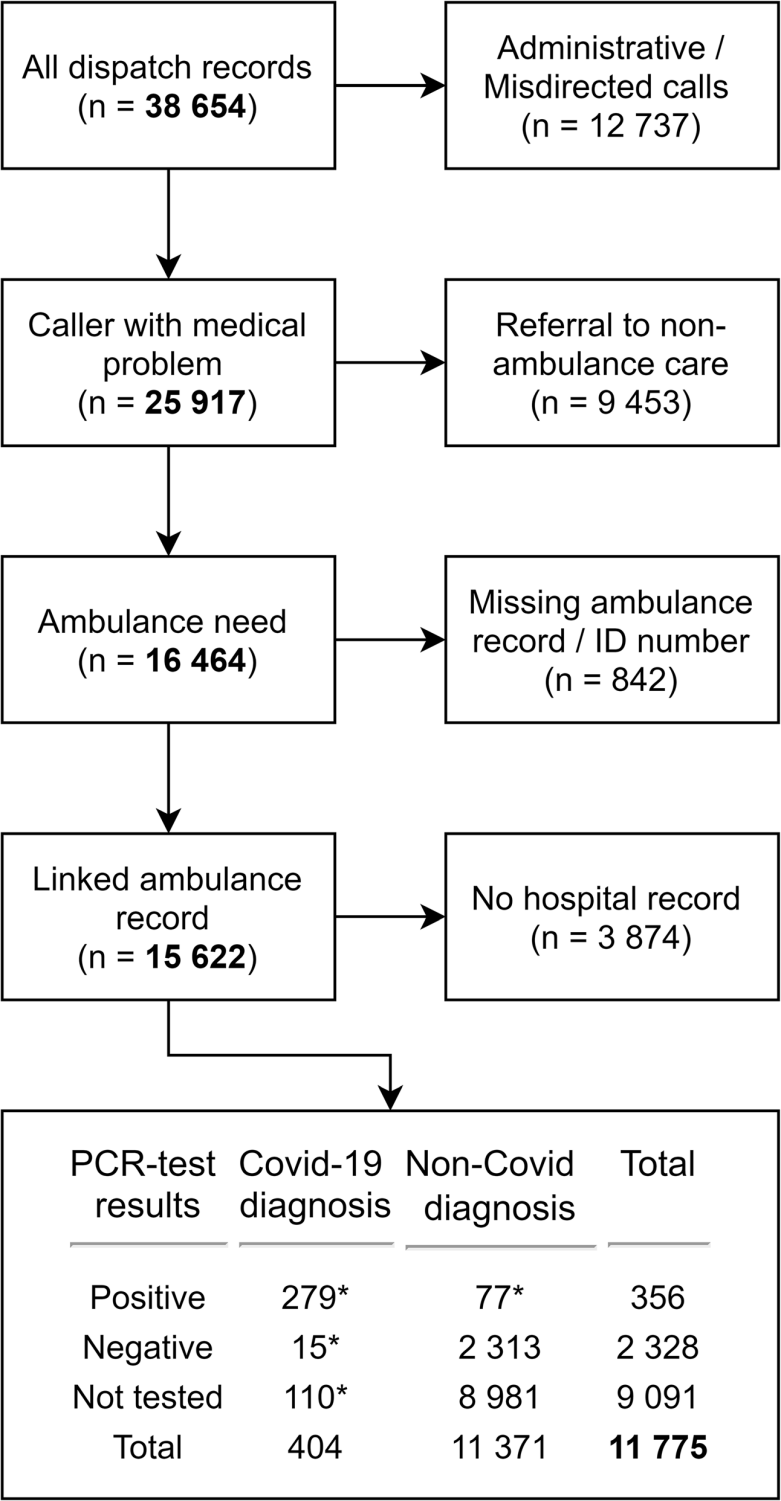
Inclusion flow chart.* Patients considered “hospital-confirmed” Covid-19

As described in Fig. 1, a total of 38,654 records were identified. Of these, 25,917 had a medical problem, with the remainder being either administrative assignments (e.g. stand-by or test records) or misdirected calls (e.g. police/fire calls or accidental calls). Of these, 16,464 were determined by a dispatch nurse to require an ambulance, with the remainder of patients referred to alternate forms of care (e.g. alternate forms of transport to definitive care, the nursing advice line, or to self-care pending a change in symptoms). Of these, 15,622 had both a documented personal identification number and a linked ambulance record. Finally, hospital records could be identified for 11,775 of these patients, with the remainder having either been treated and released on-scene (and did not visit a hospital-based care provider within 72 h), or had a misregistered personal identification number resulting in a linkage failure. Thus, a total of 11,775 hospital visits with linked prehospital data were identified over the study period.

Of the patients included, 2684 (23%) were tested for ongoing SARS-CoV-2 infection via PCR, of whom 356 (13%) were found to be positive at any point during the hospital episode. A total of 404 patients had a primary diagnosis code indicating Covid-19, of whom 125 were diagnosed with Covid-19 without a positive test (potentially due to the results of tests performed outside of the hospital, or via other means of diagnosis including radiology). An additional 77 patients were found to have a positive test result with no corresponding diagnosis, for a total of 481 “hospital-confirmed” cases of Covid-19.

Of the included records, 6794 occurred on or after April 14 and were included in the analysis of prehospital predictive value. Of 2252 PCR tests were performed following this date, 279 were positive. An additional 77 Covid-19 patients were identified via diagnosis code, for a total of 481 hospital confirmed cases during this time frame.

**Fig. 2:**
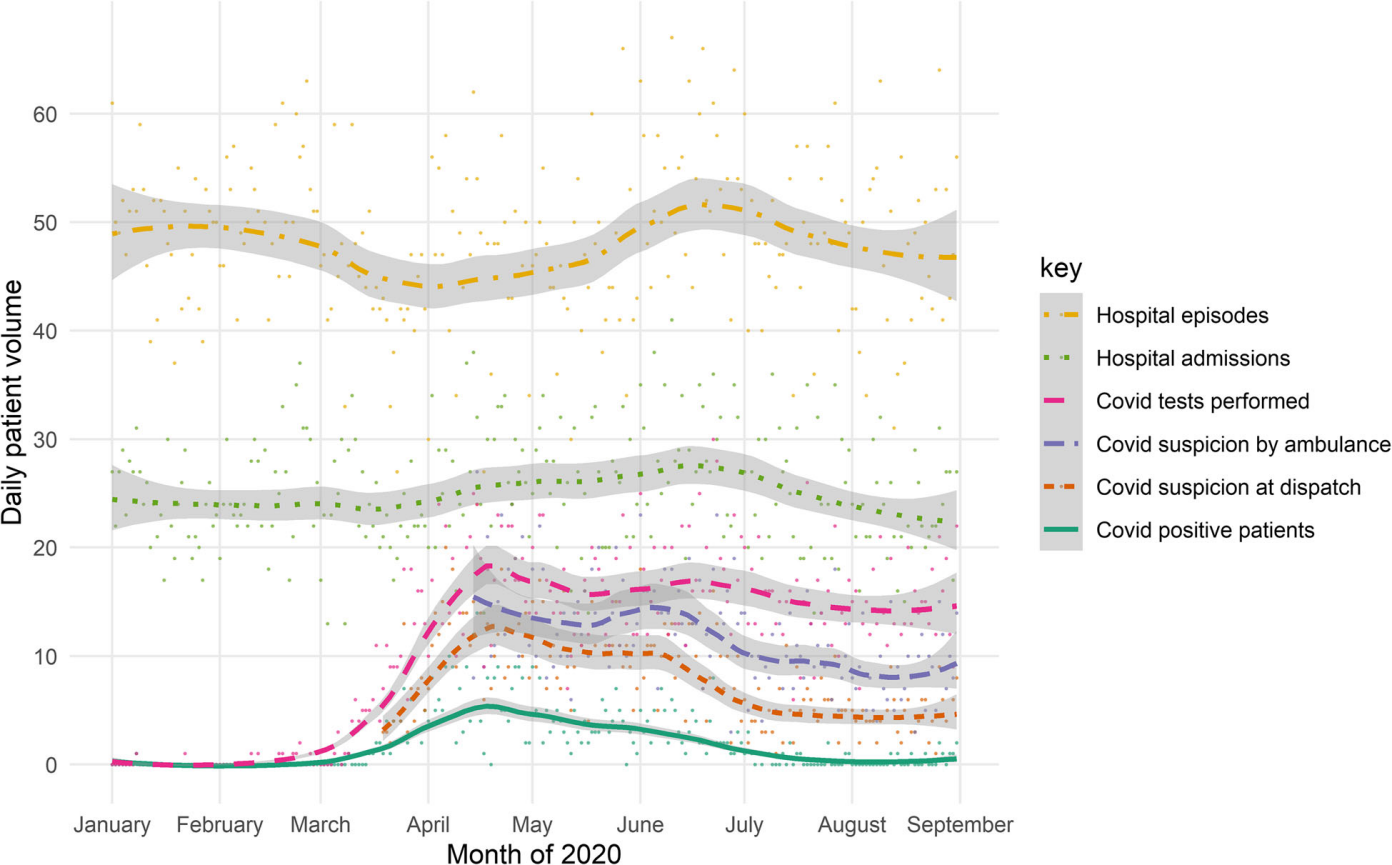
Absolute daily patient volumes (individual points) over study period with smoothed average rates with 95% confidence intervals (shaded area). Rolling averages for dispatch and ambulance suspicion are plotted from the dates they were implemented (March 19 and April 14, respectively)

A total of 1381 patients had suspicion of Covid-19 documented by the dispatch center, while 1690 had suspicion documented in the ambulance journal. The volume of these patients over time is reported in Fig. 2 below.

Table 1 below presents descriptive statistics regarding the study cohort, divided into comparison groups.

**Table 1:** Patient demographics and clinical characteristics

N	Pre-Covid	Assumed negative	Tested negative	Positive
	2998	5983	2313	481
Female, %	52.8 (51.0–54.6)	51.2 (50.1–52.5)	50.2 (48.2–52.3)	47.0 (42.2–51.1)
Age, median	70.0 (70.0–71.0)	65.0 (64.0–66.0)	77.0 (76.0–77.0)	73.0 (69.0–74.0)
High priority dispatch, %	48.1 (46.3–49.9)	46.7 (45.4–48.0)	40.9 (38.9–42.8)	30.1 (25.8–34.3)
Abnormal airway, %	2.9 (2.3–3.5)	1.9 (1.5–2.3)	3.7 (3.0–4.5)	1.3 (0.4–2.4)
Abnormal breathing, %	17.0 (15.7–18.4)	9.6 (8.8–10.3)	32.4 (30.5–34.4)	46.7 (42.3–51.2)
Abnormal circulation, %	16.8 (15.4–18.1)	12.6 (11.7–13.4)	25.3 (23.4–27.0)	26.0 (21.8–30.2)
Supplemental oxygen, %	15.3 (14.0–16.6)	7.2 (6.5–7.8)	29.5 (27.5–31.3)	36.4 (31.8–41.0)
Medication administration, %	38.6 (36.9–40.3)	32.3 (31.1–33.5)	38.3 (36.4–40.3)	28.5 (24.7–32.4)
High priority transport, %	15.4 (14.2–16.7)	11.0 (10.2–11.9)	22.0 (20.0–23.8)	14.1 (11.0–17.5)
Total mission duration (minutes), median	104 (102–105)	102 (101–103)	120 (118–122)	128 (123–132)
Admitted to hospital, %	49.3 (47.4–51.1)	37.6 (36.4–38.8)	88.6 (87.2–89.9)	83.4 (80.0–86.7)
Admitted to Intensive Care Unit, %	2.0 (1.5–2.6)	0.6 (0.4–0.8)	3.9 (3.1–4.7)	16.2 (12.9–19.5)
30-day mortality, %	5.9 (5.1–6.7)	3.4 (3.0–3.8)	11.3 (9.9–12.6)	23.7 (19.8–27.4)

All data presented with point estimates and bootstrapped 95% Confidence intervals. Grouped by patients cared for prior to the outbreak of Covid-19 from Jan 1-Feb 29 (Pre-Covid), those not tested for Covid-19 (Assumed negative), tested negative (Tested negative), and positive for Covid-19 per PCR-test and/or primary diagnosis code (Positive)

Covid-19 patients had a 30-day mortality rate of 23.7% (95% CI 19.8–27.4), and 16.2% (12.9–19.5) were admitted to an intensive care unit. 30.1% (25.8–34.3) of Covid-19 patients received a high priority ambulance from dispatch, while 14.1% (11–17.5) were transported by the ambulance with a high priority. 36.4% (31.8–41.0) received supplemental oxygen, while 28.5% (24.7–32.4) received other medications administered by ambulance crews. The median mission time (from receipt of call at the dispatch center to ambulance clearance from the hospital, including time spent sanitizing the ambulance) was 128 (123–132) minutes.

Table 2 presents the distribution of Covid-19 suspicion and test results across call types documented by the EMD center. A total of 1223 patients (18%, including 27 patients with an upper airway-related complaint) contacted the dispatch center with a primary complaint directly related to the Covid-19 suspicion guidelines, while 222 (46%) confirmed Covid-19 cases were among these callers.

**Table 2:** Covid-19 suspicion and testing rates by primary complaint at dispatch with more than 100 occurrences from April 14 onwards, ordered by descending proportion of confirmed Covid-19 cases

Primary Complaint	n	Suspicion at dispatch, %	Suspicion by ambulance, %	Tested at hospital, %	Hospital-confirmed ^a^ Covid-19, %
Infection	261	68.6 (62.8–74.3)	78.5 (73.9–83.5)	72.8 (67.4–78.2)	29.1 (23.8–34.9)
Fever	147	71.4 (64.6–79.6)	76.9 (70.1–83.7)	74.1 (67.3–81.0)	16.3 (10.9–22.4)
Difficulty Breathing	790	50.9 (47.5–54.3)	65.4 (61.8–68.5)	62.9 (59.9–66.1)	14.7 (12.3–17.1)
General Elderly^b^	344	17.2 (13.4–21.5)	29.1 (24.1–33.7)	54.7 (49.1–60.2)	6.4 (3.8–9.0)
General Adult^b^	139	28.8 (21.6–36.0)	29.5 (22.3–37.4)	38.1 (30.2–46.0)	5.8 (2.2–10.1)
Reduced consciousness	262	12.6 (8.8–16.4)	22.9 (18.3–27.9)	38.9 (33.2–45.0)	4.6 (2.3–7.3)
Fainting	117	12.0 (6.8–17.9)	19.7 (12.8–27.4)	22.2 (14.5–29.9)	3.4 (0.9–6.8)
Stroke	428	5.4 (3.5–7.5)	14.7 (11.4–18.0)	35.3 (30.8–40.2)	3.3 (1.9–5.1)
Seizure	207	4.3 (1.9–7.2)	10.6 (6.8–15.4)	20.3 (15.0–25.6)	2.4 (0.5–4.8)
Other	908	11.3 (9.1–13.4)	15.7 (13.4–18.1)	24.1 (21.3–27.1)	2.2 (1.3–3.2)
Dizziness	144	6.9 (3.5–11.8)	14.6 (9.0–20.8)	17.4 (11.1–23.6)	2.1 (0.0–4.2)
Major trauma	1203	2.8 (1.9–3.8)	8.1 (6.6–9.7)	26.4 (23.9–28.9)	1.8 (1.1–2.7)
Missing	118	0.0 (0.0–0.0)	11.0 (5.9–16.9)	23.7 (16.1–31.4)	1.7 (0.0–4.2)
Abdominal pain	548	8.4 (6.2–10.9)	17.2 (13.9–20.4)	17.9 (14.6–21.2)	1.5 (0.5–2.6)
Chest pain	813	9.5 (7.4–11.4)	17.7 (15.3–20.5)	19.7 (17.1–22.4)	1.2 (0.5–2.1)
Arrythmia	153	3.3 (0.7–6.5)	14.4 (9.2–20.3)	11.8 (6.5–17.0)	0.7 (0.0–2.6)

All data presented with point estimates and bootstrapped 95% Confidence intervals

^a^ Confirmed based on either a positive PCR-test or by hospital diagnosis code

^b^ These categories capture patients with non-specific complaints that cannot be further categorized

**Fig. 3:**
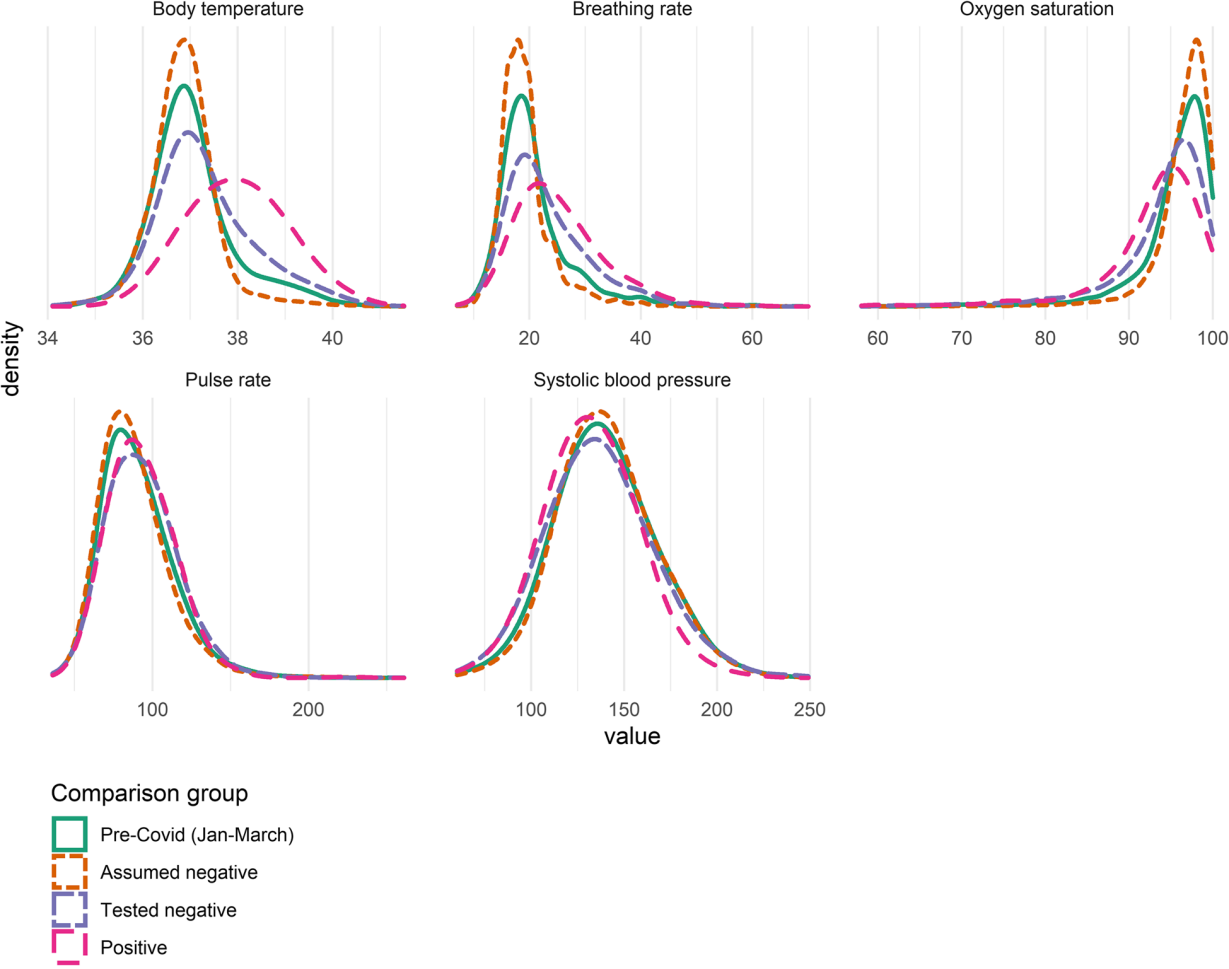
Distribution of vital signs of Covid-19 patients in relation to comparison cohorts. Note: Distribution functions have no interpretable Y-axis, and it is thus left blank. Rather, the area under each curve equals 1, and the height of the curve is inversely proportional to the variance in the data

Figure 3 presents density functions for the initial vital signs of patients in each of the comparison groups. In comparing Covid-19-positive and patients tested negative, all vital signs except for pulse rates demonstrated statistically significant differences in central tendency per Wilcoxon rank sum tests. Body temperature demonstrated the most substantial effects, with 23% of patients with a negative Covid-19 test result presenting with a fever of > = 38 degrees centigrade, and 51% of Covid-19 positive patients presenting with this degree of fever upon contact with the ambulance.

### Prehospital assessments

Table 3 below presents the overall predictive values for dispatch and ambulance suspicion of Covid-19. Given the risk of misclassification-bias due to low testing rates, data are presented both for the full cohort of patients, and for only those with a documented PCR-test for SARS-CoV-2. The prevalence of SARS-CoV-2 in the full population was 5, and 11% among tested patients. The apparent prevalence of Covid-19 (i.e., the percentage of patients with documented suspicion) in the full population was 17% at the dispatch, and 25% in the ambulance. Apparent prevalence among tested patients was 32 and 49% at the dispatch and ambulance, respectively. In the full population, the sensitivity of documented Covid-19 suspicion at dispatch was 75.9% (71.0–80.3) with a specificity of 86.4% (85.5–87.2) in the full cohort. The sensitivity of the ambulance suspicion was 82.2% (77.8–86.1), with a specificity of 78.2% (77.2–79.2). Levels of specificity and negative predictive value were lower within the cohort of tested patients only.

**Table 3:** Predictive value of dispatch and ambulance suspicion of Covid 19 among all patients, and tested patients only (April 14 – August 31)

	All patients (*n* = 6776)		Tested patients only (*n* = 2252)	
	Dispatch	Ambulance	Dispatch	Ambulance
Apparent prevalence, %	16.8 (15.9–17.7)	24.9 (23.9–25.9)	31.9 (30.0–33.9)	49.3 (47.2–51.4)
True prevalence, %	5.1 (4.6–5.7)	5.1 (4.6–5.7)	11.1 (9.8–12.4)	11.1 (9.8–12.4)
Sensitivity, %	75.9 (71.0–80.3)	82.2 (77.8–86.1)	71.1 (65–76.6)	79.1 (73.5–84.0)
Specificity, %	86.4 (85.5–87.2)	78.2 (77.2–79.2)	73.0 (71.0–74.9)	54.4 (52.2–56.6)
Positive predictive value, %	23.1 (20.7–25.7)	16.9 (15.2–18.8)	24.7 (21.5–28)	17.7 (15.5–20.1)
Negative predictive value, %	98.5 (98.2–98.8)	98.8 (98.4–99.1)	95.3 (94.1–96.3)	95.4 (94.1–96.6)

All data presented with point estimates and exact binomial 95% Confidence intervals

**Fig. 4:**
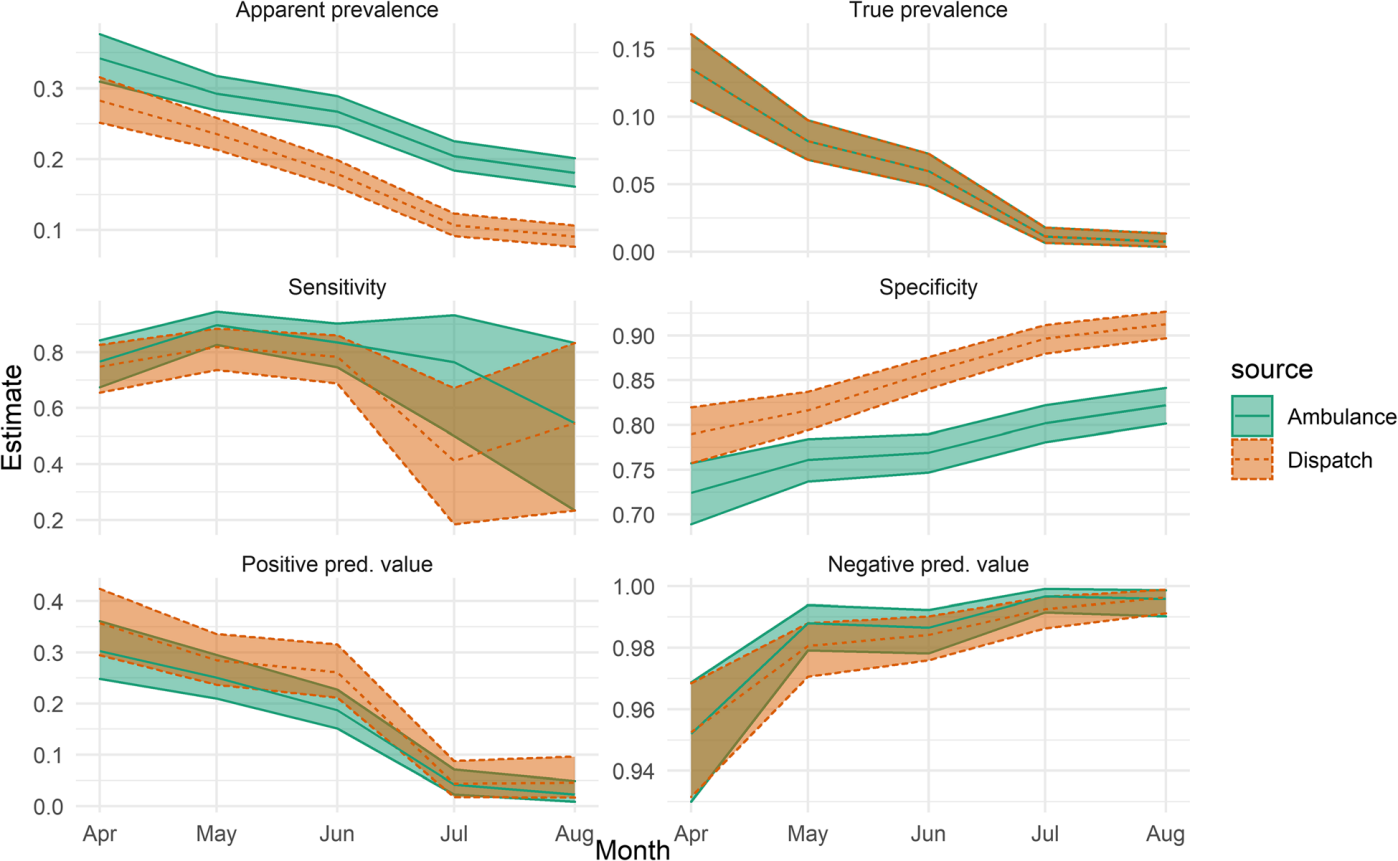
Predictive value of Dispatch and Ambulance suspicion among all patients (*n* = 6776) per month, including 95% confidence intervals

Figure 4 presents this data per study month. Over time, both the apparent and true prevalence of Covid-19 declined (Note that structured documentation on the ambulance was implemented at the peak of the pandemic). Specificity and negative predictive value increased while positive predictive value declined substantially over time. Between April and May, dispatch sensitivity changed from 75% (65–83) to 82% (74–88), while ambulance sensitivity changed from 77% (67–84) to 89% (83–95), while in later months, the scarcity of true positive cases resulted in wide confidence intervals (Fig. 4).

## Discussion

### Key results

In this study, we found that the 76% of hospital-confirmed Covid-19 patients were identified by dispatch nurses, while 82% were identified by ambulance nurses. We identified a relatively high 30-day mortality rate of 24% among Covid-19 patients, but a relatively low rate of emergent response (30%) and transport (14%). Covid-19 patients presented with a diverse set of complaints, and a majority of Covid-19 patients (54%) presented with primary complaints not typical of Covid-19. With the exception of body temperature, initial vital signs had little predictive value for the identification of Covid-19 patients.

### Limitations

This retrospective analysis has several important limitations. Documentation completeness issues likely entail an under-estimation of the true sensitivity of prehospital suspicion of Covid-19, given that suspicion is often likely to be communicated upon handover between care providers verbally, or in free-text notes. We chose to limit this analysis to structured documentation (i.e., the selection pre-defined options) however, as only this form of data has the potential to be used in automated alerting and monitoring systems.

We found that overall testing rates among patients arriving at the hospital was low (23%), constituting a potential source of misclassification bias. Furthermore, testing was unequally distributed, with for instance 52% of patients admitted to the hospital tested for ongoing SARS-CoV-2 infection, but only 8% of patients discharged from the ED being tested. Some portion of these untested patients were in all likelihood asymptomatic or mildly symptomatic carriers of SARS-CoV-2 [18]. While false negative patients in this group are unlikely to have clinically severe Covid-19-related symptoms, they nonetheless represent a disease vector which must be identified and isolated to prevent contagion. Further misclassification may result from errors in the tests themselves, though the combined oro-nasopharyngeal sampling method employed in the region is considered to be perhaps the most sensitive available sampling approach for the diagnosis of ongoing SARS-CoV-2 infection [19].

To address the risk of misclassification-bias, we performed a sensitivity analysis investigating predictive value within only the cohort of patients whose SARS-CoV-2 status was confirmed via PCR testing. Specificity was significantly lower in the sensitivity analysis, likely owing to the exclusion of a large number of negative cases. Estimates of sensitivity also tended to be lower in this analysis, but the difference could not be confirmed statistically. This loss of fidelity among patients identified via testing could be due to the inclusion of a larger proportion of patients with asymptomatic or mildly symptomatic SARS-CoV-2 infections, and/or the exclusion of clinically relevant positive cases identified by means other than PCR-testing.

The inclusion criteria for this study select for patients cared for through the typical prehospital care pathway of ambulance transport to the ED, and many patients referred to alternate forms of care by dispatchers and ambulance crews, as is common in the studied region [20], were not included owing to the lack of relevant outcome data for these patients. It may also be that some cases of Covid-19 were hospital-acquired, and that prehospital care providers could not have been expected to identify them. The data from this single-center study should be considered together with data collected in other settings to form a more general picture of the clinical impact of Covid-19 in the domain of prehospital care.

### Interpretation

While the volume of Covid-19-related calls peaked in mid-April, the number of hospital episodes and admissions remained stable, owing to an increase in the number of patients directed by dispatchers and ambulance crews to non-ED destinations. In terms of pre-hospital acuity, Covid-19 patients had the lowest proportion of high priority (i.e., lights and sirens) dispatch responses of any comparison group (30%), and similar levels of high priority transport compared to the baseline population (14%). Expressed another way, the prevalence of Covid-19 did not differ between patients transported with a high- or low priority. Covid-19 patients were most likely to have signs of abnormal breathing (47%), and to receive supplemental oxygen (36%), but least likely to receive prehospital medications (28%) compared with other patient cohorts. Outcomes for Covid-19 patients were substantially worse than any other cohort, with 16% of patients cared for in the ICU, and a 30-day mortality rate of 23%.

It should be noted that prehospital priority was associated with patient outcomes in all comparison groups, as found in previous studies [21]. Within the cohort of Covid-19-positive patients, the 30-day mortality rate of Covid-19 patients transported to the hospital with lights and sirens was for instance 40%, compared with 21% for patients transported with a low priority. These findings thus suggest a discrepancy between prehospital prioritization and outcomes, and not a complete lack of association. This discrepancy could point to a difficulty in recognizing the early symptoms of severe Covid-19 infections, or to a more rapid deterioration among these patients after handover to definitive care.

Dispatch nurses were more selective in identifying suspected Covid-19 cases than their counterparts on the ambulance, with overall suspicion rates of 16% vs. 24%. This resulted in a higher specificity (87% vs 79%), but a lower sensitivity (76% vs 82%). The negative predictive value of prehospital suspicion shifted from 95% at the beginning of the pandemic to 99% in the latter phases, likely due in large part to changes in the overall prevalence of Covid-19 in the population. A priori, we suspected that we might find an increasing trend in predictive value over time due to improved guidelines and care providers learning more about the presentation of Covid-19 patients. While we did observe a small initial uptick in sensitivity from April to May, we cannot confirm the existence of such an effect with a sufficient level of confidence based on these observational data, and this should be examined in further studies in other contexts.

While the level of predictive value found in this study is useful for the purposes of for instance system-level monitoring, the authors consider the level of sensitivity found here to fall short of the level required to rule out the need for isolation precautions on an individual basis. These results suggest that during periods of significant community transmission of Covid-19, provider suspicion alone is not sufficient to rule out the potential for contagion, and some level of isolation precautions should be adopted when interacting with all patients.

Our results demonstrate that roughly half of Covid-19 positive patients present with primary complaints associated with Covid-19 (infection, fever, upper airway complaints, or difficulty breathing), while significant numbers of Covid-19 patients present to prehospital care providers with non-typical complaints and may be difficult for care providers to identify. Simultaneously, only a minority of patients presenting with “typical” complaints were indeed later diagnosed with Covid-19. Previous studies have employed the documentation of a Covid-19-associated prehospital primary complaint as a proxy measure for the impact of Covid-19 at the system-level [8, 9], and these results cast some doubt on the validity of this approach of gauging the true impact of Covid-19.

While patient vital signs demonstrated a limited capacity to detect the presence of SARS-CoV-2, they are likely to play a more substantial role in identifying severe cases with poor predicted outcomes [21–23]. The low overall testing rates and the preference towards testing higher-acuity patients found in this study entail a substantial risk for non-random misclassification-bias which would be imparted to models seeking to identify Covid-19 positive patients within a general population where testing is not widespread or randomly distributed [24]. Approaches for mitigating such systematic misclassification bias in testing should be investigated in further research.

### Generalizability

Our results were in line with a previous investigation by Fernandez et al. of the sensitivity of Covid-19 suspicion documented in free-text notes written by ambulance crews, but given the substantial difference in overall prevalence of Covid-19 between these studies (5% here vs. 1% in Fernandez et al.) comparisons should be made only with caution, particularly with regards to specificity [11]. In examining the characteristics of Covid-19 patients presenting to the prehospital care system in this region, we found a 24% rate of 30-day mortality, which is congruent with preliminary investigations of hospitalized patients diagnosed with Covid-19 in Denmark, Norway, and Italy [23, 25, 26]. The dispatch and ambulance personnel investigated here consisted of nurses with 3–4 years of formal education, which is a relatively high level of education by international standards. The idiosyncratic approach taken by Swedish public health authorities [27], the 23% overall testing rate, and 11% test positivity rate should also be considered when generalizing these results to other settings.

## Conclusion

While prehospital suspicion was strongly indicative of hospital-confirmed Covid-19, based on the sensitivity identified in this study, prehospital suspicion should not be relied upon as a single factor to determine the need for isolation precautions on the ambulance or at the emergency department. The descriptive analysis revealed that Covid-19 patients presented with a diverse set of primary complaints. Despite a strikingly high rate of 30-day mortality and ICU utilization, Covid-19 patients had a relatively low level of perceived prehospital acuity. The data provided may be used to improve guidelines for identifying Covid-19 patients in the prehospital setting.

## Data Availability

The datasets analyzed in the current study are available from the corresponding author and/or the Uppsala regional ambulance service (ambulanssjukvard@akademiska.se) on reasonable request.

## Abbreviations

Covid-19: Novel Coronavirus Disease 2019
SARS-CoV-2: Severe Acute Respiratory Syndrome Coronavirus 2
EMD: Emergency Medical Dispatch
PCR: (real-time,reverse transcription) polymerase chain reaction
